# Denosumab Chemotherapy for Recurrent Giant-Cell Tumor of Bone: A Case Report of Neoadjuvant Use Enabling Complete Surgical Resection

**DOI:** 10.1155/2013/496351

**Published:** 2013-07-30

**Authors:** Amit Agarwal, Brandon T. Larsen, Lawrence D. Buadu, Jack Dunn, Russell Crawford, Jonathan Daniel, Maria C. Bishop

**Affiliations:** ^1^Department of Medicine, Southern Arizona Veterans Affairs Health Care System, 3601 S 6th Avenue, Tucson, AZ 85723, USA; ^2^Department of Pathology, Mayo Clinic, Rochester, MN 55905, USA; ^3^Department of Radiology, Southern Arizona Veterans Affairs Health Care System, Tucson, AZ 85723, USA; ^4^Department of Neurosurgery, Southern Arizona Veterans Affairs Health Care System, Tucson, AZ 85723, USA; ^5^Department of Pharmacy, Southern Arizona Veterans Affairs Health Care System, Tucson, AZ 85723, USA; ^6^Department of Thoracic Surgery, Providence Portland Medical Center, OR 97213, USA

## Abstract

Giant-cell tumor of the bone (GCTB) is a rare neoplasm that affects young adults. The tumor is generally benign but sometimes can be locally aggressive. There are no standardized approaches to the treatment of GCTB. Recently, the RANKL inhibitor denosumab has shown activity in this tumor type. We present the case of a young female who presented with locally advanced disease and was successfully managed with the neoadjuvant use of denosumab allowing for surgical resection of the tumor that was previously deemed unresectable. Following surgery, the patient is being managed with continued use of denosumab as ‘maintenance,' and she continues to be free of disease. Our case highlights a novel approach for the management of locally advanced and aggressive giant cell tumor of the bone.

## 1. Introduction

Giant-cell tumor of bone (GCTB) is a relatively rare neoplasm that affects young adults [1, 2]. It is a benign but locally aggressive skeletal neoplasm that causes significant bone destruction and has a predilection for the epiphyseal/metaphyseal region of long bones and the spine [2, 3]. Despite the generally benign nature of the disease, GCTBs can have highly variable and unpredictable behavior. Although there are no randomized clinical trials to assess treatment, patients are treated with surgery, radiotherapy, and occasionally systemic therapy [3]. Histologically, GCTB is characterized by abundant osteoclast-like giant cells and their precursors that express receptor activator of nuclear factor kappa-B ligand (RANKL) which is a key mediator of osteoclast activation [4–7]. RANKL signaling has been shown to have an important role in the pathogenesis of giant-cell tumors. Denosumab, a novel monoclonal antibody directed against RANKL that is currently FDA-approved for treatment of osteoporosis, has also been found to be active in GCTB and is now in clinical trials as a novel treatment for this tumor [6, 8]. We present a case of a recurrent GCTB that was initially unresectable but displayed a marked response to combination radiation and denosumab treatment, eventually enabling complete resection.

## 2. Clinical Presentation

**Figure 1:**
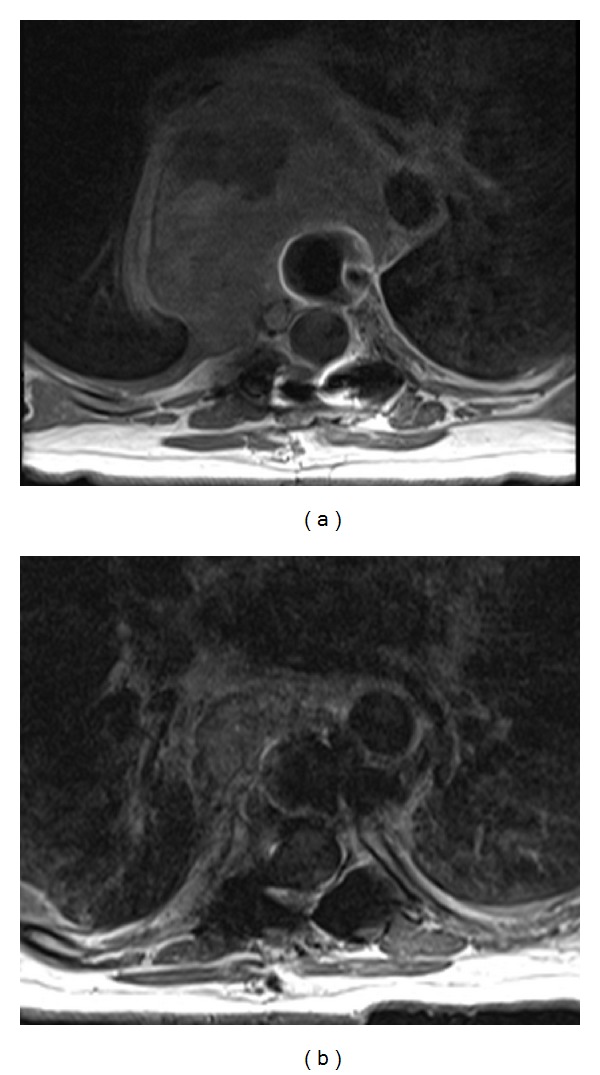
Axial T1 weighted postcontrast computed tomographic images show a large mid-thoracic spine mass (a) before and (b) 10 months after denosumab therapy.

**Figure 2:**
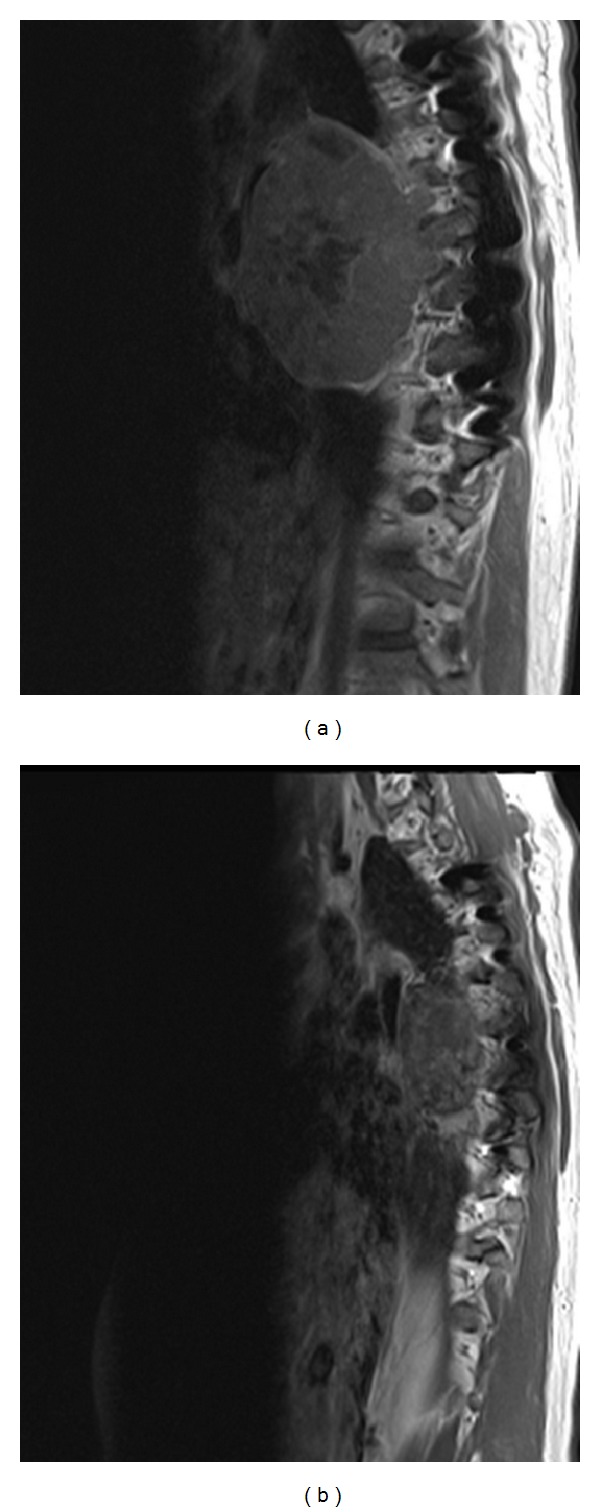
Sagittal T1 postcontrast computed tomographic images show the large mid-thoracic spine mass (a) before and (b) 10 months after denosumab therapy.

**Figure 3:**
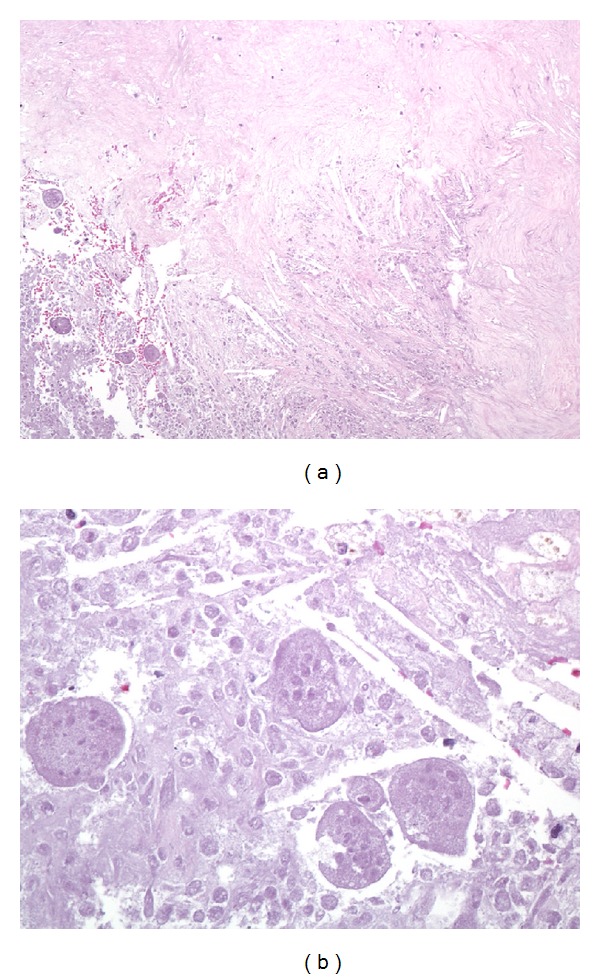
Representative photomicrographs of the paraspinal mass after denosumab therapy. At low magnification ((a); 100x, hematoxylin and eosin), extensive necrosis is apparent with abundant cholesterol clefts (center) and surrounding fibrosis (top). Residual tumor is focally recognizable but is entirely necrotic (lower left), and no viable tumor is present. At high power ((b); 400x, hematoxylin and eosin), “ghosts” of necrotic multinucleated osteoclastic giant cells are present among necrotic mononuclear tumor cells.

**Figure 4:**
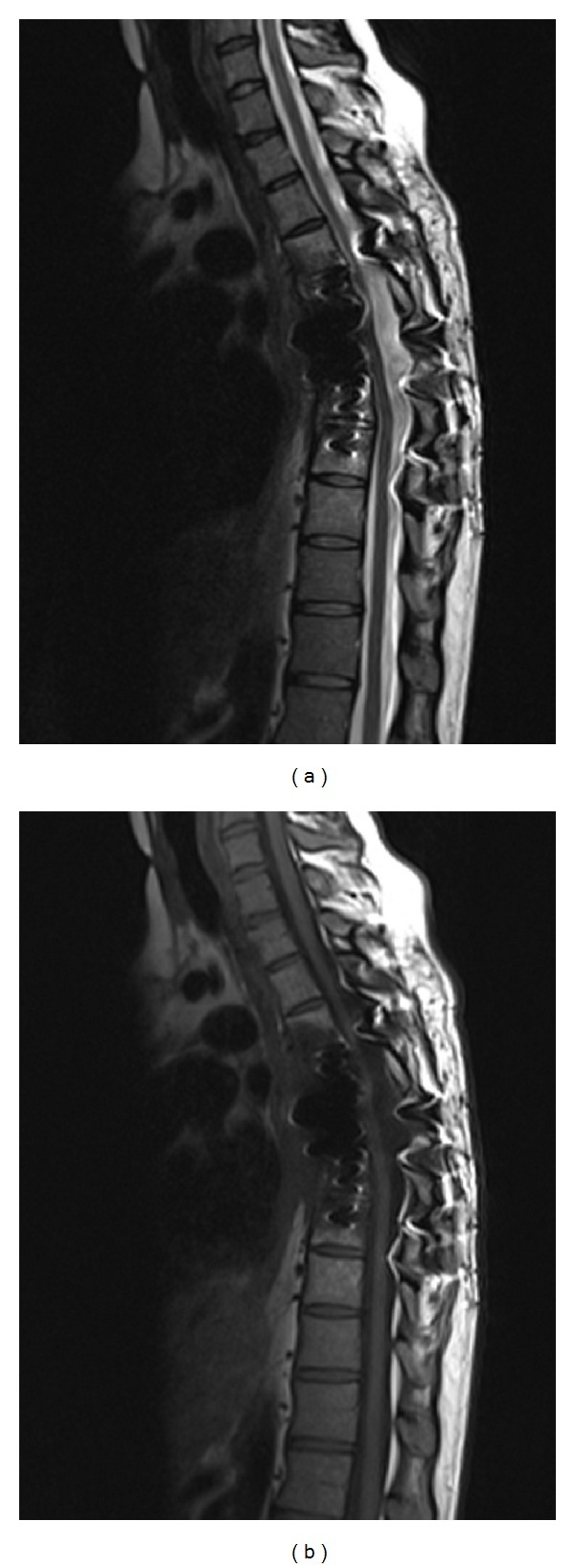
Sagittal T1 (a) and T2 (b) weighted images show postsurgical changes with no significant residual tumor 7 months after resection.

A 27-year-old woman presented with complaints of back pain in 2007 while living in Japan. Initial imaging revealed a T6 vertebral mass, and biopsy confirmed a diagnosis of GCTB. She underwent resection of the tumor with spinal stabilization. The patient was then lost to followup. In January 2010, she presented again with a 10.3 cm paraspinal mass in the T6 area on surveillance magnetic resonance imaging (MRI). This mass pushed the upper portion of the lungs posteriorly, the carina anteriorly, and the thoracic aorta laterally. Recurrent GCTB was confirmed by fine needle aspiration and biopsy. A resection was planned, and the patient was taken to surgery. Unfortunately, the tumor was unresectable due to extensive vertebral and vascular involvement. The patient was referred to radiation oncology and underwent intensity-modulated radiation therapy (5040 cGy) to the mass from March to April 2010. Follow-up computed tomographic imaging in May 2010 demonstrated an increase in the size of the mass from 9 × 8.9 cm AP to 10.2 × 9.2 cm (Figures 1(a) and 2(a)). The patient was then started on monthly denosumab in July 2010, with no major complications, and showed marked treatment response on serial imaging studies. By May 2011, the tumor had shrunk to 1.9 × 5.4 cm (Figures 1(b) and 2(b)). Three months later, the mass was found to be stable, and a repeat attempt at surgical resection was planned. In September 2011, she underwent thoracotomy with resection of the right lower lobe of the lung and removal of lateral portions of the T5–T8 vertebrae, along with the costal heads. Postoperative pathology showed a complete chemotherapeutic response, with extensive necrosis and fibrosis throughout the entire specimen and only focally recognizable tumor remaining, all of which was necrotic (Figures 3(a) and 3(b)). No viable tumor was identified. The patient had a slow but uneventful postoperative recovery. She remained free of recurrence, and an MRI done in April 2012 showed only postsurgical changes with no evidence of disease recurrence (Figures 4(a) and 4(b)). On the most recent CT imaging done in March 2013, there is no evidence of disease recurrence. The denosumab is now being administered every 3 months. The patient continues to tolerate the treatment without any adverse effects. 

## 3. Conclusion

Although GCTB is a benign neoplasm, recurrent GCTB can behave in an aggressive fashion and can be difficult to surgically resect or to treat with standard chemoradiation. Our case not only highlights this difficulty, but it also underscores the importance of multimodality management in the treatment of this tumor. In particular, our experience provides additional evidence that denosumab may be particularly useful in the neoadjuvant setting. At this time, there are no randomized studies that can help direct therapy for recurrent GCTB. The discovery of the role of RANKL signaling in the pathogenesis of GCTBs has ushered in a new treatment era for this neoplasm, and phase II studies have shown clinical benefit from the use of the RANKL inhibitor denosumab. In our case of a recurrent GCTB that was not only unresectable but also nonresponsive to traditional radiotherapy, denosumab treatment markedly shrank the tumor and enabled complete surgical resection. To date, she remains recurrence-free, while continuing to receive denosumab. This remarkable case is encouraging, and hopefully ongoing clinical studies will better define the role of anti-RANKL biologics in the multimodality management of this unusual neoplasm.
